# GWAS in people of Middle Eastern descent reveals a locus protective of kidney function—a cross-sectional study

**DOI:** 10.1186/s12916-022-02267-7

**Published:** 2022-03-01

**Authors:** Siham A. Mohamed, Juan Fernadez-Tajes, Paul W. Franks, Louise Bennet

**Affiliations:** 1 Lund University Diabetes Center, Lund University, Malmö, Sweden; 2 Department of Clinical Sciences, Lund University, Malmö, Sweden; 3 Clinical Research and Trial Center, Lund University Hospital, Lund, Sweden

**Keywords:** Genome-wide study, Chronic kidney disease, Estimated glomerular filtration rate, Middle East, Type 2 diabetes

## Abstract

**Background:**

Type 2 diabetes is one of the leading causes of chronic kidney failure, which increases globally and represents a significant threat to public health. People from the Middle East represent one of the largest immigrant groups in Europe today. Despite poor glucose regulation and high risk for early-onset insulin-deficient type 2 diabetes, they have better kidney function and lower rates of all-cause and cardiovascular-specific mortality compared with people of European ancestry. Here, we assessed the genetic basis of estimated glomerular filtration rate (eGFR) and other metabolic traits in people of Iraqi ancestry living in southern Sweden.

**Methods:**

Genome-wide association study (GWAS) analyses were performed in 1201 Iraqi-born residents of the city of Malmö for eGFR and ten other metabolic traits using linear mixed-models to account for family structure.

**Results:**

The strongest association signal was detected for eGFR in *CST9* (rs13037490; *P* value = 2.4 × 10^−13^), a locus previously associated with cystatin C-based eGFR; importantly, the effect (major) allele here contrasts the effect (minor) allele in other populations, suggesting favorable selection at this locus. Additional novel genome-wide significant loci for eGFR (*ERBB4*), fasting glucose (*CAMTA1*, *NDUFA10*, *TRIO*, *WWC1*, *TRAPPC9*, *SH3GL2*, *ABCC11*), quantitative insulin-sensitivity check index (*METTL16*), and HbA1C (*CAMTA1*, *ME1*, *PAK1*, *RORA*) were identified.

**Conclusions:**

The genetic effects discovered here may help explain why people from the Middle East have better kidney function than those of European descent. Genetic predisposition to preserved kidney function may also underlie the observed survival benefits in Middle Eastern immigrants with type 2 diabetes.

**Supplementary Information:**

The online version contains supplementary material available at 10.1186/s12916-022-02267-7.

## Background

Chronic kidney disease (CKD) is a leading public health problem, affecting more than 13% of the world’s population [1]. Along with aging, obesity, and hypertension, type 2 diabetes (T2D) is one of the leading causes of CKD [2], with 1:10 deaths in people with diabetes attributed to kidney failure [3].

CKD is caused by a gradual loss of kidney function characterized by diminished glomerular filtration rate (GFR) and/or other markers of kidney damage [4]. Variants in genes like *UMOD*, *GPX1*, *GSTO1*, *GSTO2*, *SHROOM3*, and *MGP* have been associated with susceptibility to CKD [5]. The prevalence of CKD varies between ethnicities, with African Americans at particularly high risk [6, 7].

The city of Malmö is multicultural, hosting people from approximately 180 countries. Every third citizen is born abroad, with the largest immigrant group born in Iraq and representing 3.4% of the total population [8]. The MEDIM cohort (the impact of Migration and Ethnicity on Diabetes in Malmö) is a population-based cohort comprised of people aged 30 to 75 years, born in either Iraq or Sweden, and resident in Malmö. Data from the MEDIM study has revealed that Iraqi migrants in Malmö are at twice the risk of T2D [9] and have younger age of disease onset compared with the Swedish born population, with high burden of diabetes family history, and poorer glycemic control and insulin sensitivity [10]. Paradoxically, despite the poorer metabolic health profile, Iraqi migrants present with better kidney function and lower blood pressure than their Swedish counterparts [11], rendering this a particularly interesting cohort for genetic studies of these traits.

In the last decade, GWAS has improved the understanding of the genetic architecture of polygenic diseases such as CKD and T2D [12]. However, these studies were performed predominantly in European ancestry populations, which may limit generalizability of findings to other ethnicities, underscoring the need for greater ethnic diversity in GWAS [13]. Regardless of trait, no published GWAS has been performed in cohorts of Iraqi ancestry.

The purpose of this study was to undertake GWAS of eGFR and 10 additional diabetes-related traits in immigrants of Iraqi ancestry from the MEDIM cohort.

## Methods

### Study participants and phenotyping

The phenotyping process has been described in detail previously [9, 14]. Briefly, after signing informed consent, participants aged 30 to 75 years were randomly selected from the Malmö census register. Individuals with severe physical or mental illness were excluded. The final cohort included here comprised 1201 men and women born in Iraq with complete GWAS data (see Additional file 1: Fig S1).

Assessments were undertaken from February 1, 2010, through December 31, 2012. Participants were invited to a health exam and were told not to eat or drink anything besides water and not to utilize tobacco from 10 pm the day preceding testing; they were also asked to record their current medication. A standard physical checkup was performed prior to sample collection. Clinical variables such as waist circumference, height, weight, body mass index (BMI), systolic blood pressure (SBP), and diastolic blood pressure (DBP) were assessed. DNA for genotyping was extracted from buffy coat. Blood samples were collected when participants were fasting and during a 75-g oral glucose tolerance test (OGTT) from which insulin sensitivity index (ISI), corrected insulin response (CIR), and oral disposition index (DIO) were calculated [15]. Blood glucose, homeostasis model assessment of β cell function (HOMA-β), glycated hemoglobin (HbA1C), quantitative insulin sensitivity check index (QUICKI), and estimated glomerular filtration rate (eGFR) were assessed in fasting samples. eGFR was calculated based on the mean of eGFR creatinine and eGFR cystatin C. A detailed overview of how eGFR was calculated is provided elsewhere [11]. QUICKI and HOMA-β were computed as follows:

$$QUICKI=1/\left[\mathit{\log}(FI)+\mathit{\log}(FG)\right]$$

$$HOMA-\beta =20\times FI/ FG-3.5,$$

where FG and FI denotes fasting glucose (mmol/L) and fasting insulin (mIE/L) respectively.

### Statistics

#### Genotyping and quality control

Genotyping was performed at the Swedish National Genomics Infrastructure - SciLifeLab (Uppsala, Sweden) using the Infinium assay (Illumina, USA), and data were analyzed using GenomeStudio 2.0.3 (Illumina, USA) [16]. The genome build used for genotype curation was 37 (GRCh37). A total of 759,993 SNP markers were analyzed with a genotyping call rate of 99.26%.

Phenotype data were log-transformed to approximate a normal distribution. SNPs and individuals with low genotype calls were excluded using a threshold of 0.02. Sex discrepancy was checked based on X chromosome heterozygosity, and, where necessary, sex was determined using genotype. SNPs with a minor allele frequency (MAF) < 0.01 were removed owing to insufficient samples size, as were SNPs deviating from Hardy-Weinberg equilibrium (*p* value < 1 × 10^−6^). Individuals with a heterozygosity rate deviating more than 3 SD from the sample mean were also excluded. Duplicate individuals and cryptic relatedness among samples were checked using PLINK’s π-hat pairwise identity by descent (IBD) estimate, calculated as follows:

$$\pi - hat=\mathrm{P}\ \left( IBD=2\right)+0.5\times \mathrm{P}\ \left( IBD=1\right)$$

where *P* represented probability

A threshold of π-hat > 0.2 was used to classify pairs as being related. Duplicate individuals (22 individuals) with a π-hat = 1 were removed. A total of 1201 individuals and 482 959 SNPs survived quality control (Additional file 1: Fig S1).

Population stratification was checked using the multidimensional scaling (MDS) approach. To check for possible outliers, an MDS plot was generated anchored to the 1000 Genomes dataset of known ethnic background. The first seven MEDIM MDS were retained and used as covariates for the association analysis. The standard quality control steps mentioned above and control for population stratification was performed using PLINK version 1.9 (an open-source whole genome association analysis toolset), and R-version 4.1.0 was used for generating plots.

#### Imputation

The quality-controlled genotype data was prepared for imputation using the Haplotype Reference Consortium (HRC) preparation checking tool. Imputation was performed via Michigan Imputation Server (www.imputationserver.sph.umich.edu) executed by minimac4 algorithm and European HRC panel. Phasing was implemented by Eagle v2.4. Following imputation, SNPs were filtered based on imputation info (*R*^2^ ≥ 0.3) and MAF > 0.01.

#### Association analysis

The associations between the 7,743,666 genotyped and imputed SNPs and the 11 quantitative traits were analyzed using linear mixed models. The 11 traits were fasting glucose, HOMA-β, HbA1C, BMI, CIR, ISI, DIO, QUICKI, SBP, DBP, and eGFR. Age, sex, and the first seven MDS principal components were included as covariates. The association analyses were conducted using BOLT-LMM software [17]. By default, BOLT-LMM assumes a Bayesian mixture-of-normal prior for the random effect attributed to SNPs other than the one being tested. This random effect reflects the polygene background and environmental effect that could affect the calculation of genetic association. Example of these random effects could be cryptic relatedness or population structure.

A conventional genome-wide significance threshold of *P* < 5.0 × 10^− 8^ was used. After the association analysis, significant SNPs were clumped to determine the lead representative SNP within a 250-kb LD block. Clumping was done using the integrative web-based platform, FUMA (https://fuma.ctglab.nl/) [18]. The variant effect for the lead SNP was predicted using Ensembl Variant Effect Predictor (VEP) [19]. Results from this analysis were cross-referenced with the NHGRI-EBI GWAS Catalog to identify previously reported signals [20].

#### Replicates

Not applicable

#### Key resources

The following RRIDs tools and dataset were utilized in the GWAS:

- Functional Mapping and Annotation of Genome Wide Association Studies, RRID:SCR_017521
- PLINK, RRID:SCR_001757
- R Project for Statistical Computing, RRID:SCR_001905
- Michigan Imputation Server, RRID:SCR_017579
- GWAS: Catalog of Published Genome-Wide Association Studies, RRID:SCR_012745
- 1000 Genomes: A Deep Catalog of Human Genetic Variation, RRID:SCR_006828
- SAMtools/BCFtools, RRID:SCR_005227

## Results

A European reference panel was used for imputation owing to the unavailability of Arab-ancestry haplotype reference panels. Of the available ancestral reference panels, the MEDIM cohort aligns most closely with the European-ancestry reference panel (Additional file 1: Fig S2).

In analyses assessing the degree of relatedness among MEDIM participants, 431 individual pairs with a π-hat value > 0.2 were found. Out of these individuals, 22 pairs were identified as duplicate individuals. For each duplicate pair, the observation with the lowest genotyping call rate was removed. In a histogram, the number of related MEDIM participants is presented (Additional file 1: Fig S3).

The mean age of the MEDIM cohort was 46.2 years, and the majority was male (60.5%). The average eGFR in the study population was 89.8 ml/min per 1.73m^2^. The characteristics of study participants are shown in Additional file 2: Table S1.

**Fig. 1:**
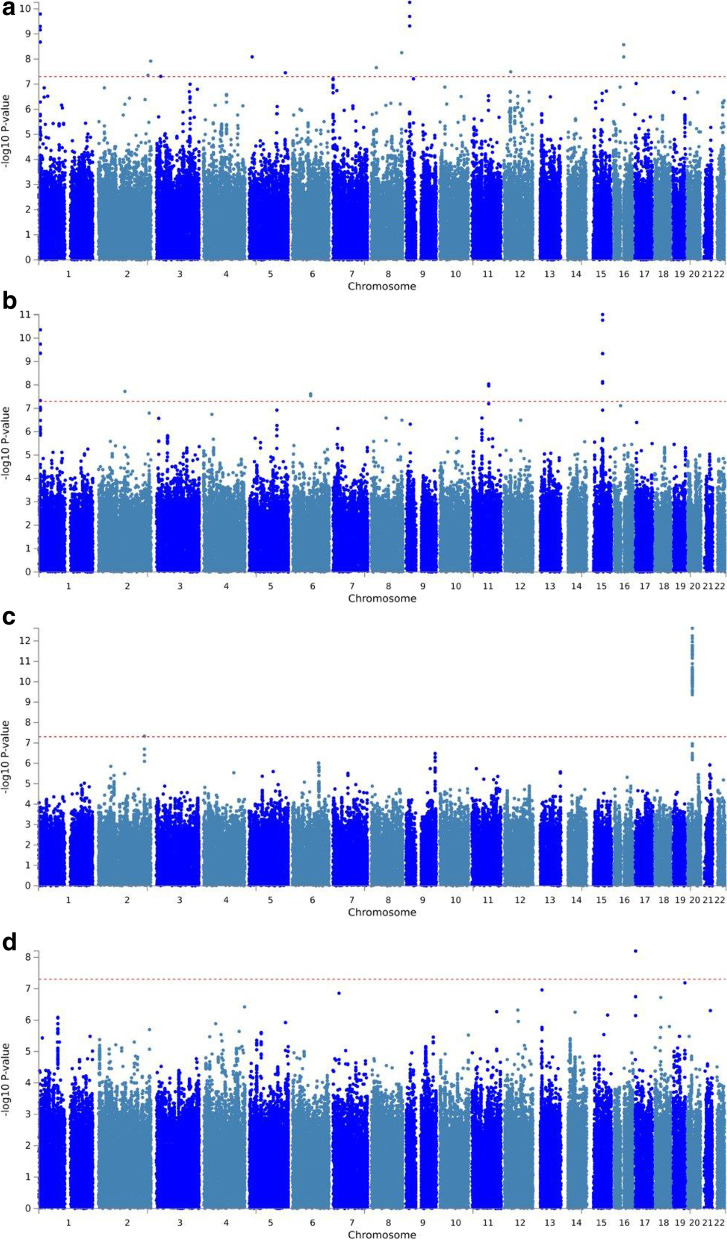
Manhattan plots for the association of SNPs with **A** fasting glucose, **B** HbA1C, **C** eGFR, and **D** QUICKI. Graph displays −log10 *P* values (*y*-axis) against chromosomal location (*x*-axis). The red line indicates genome-wide significance threshold (*P* value = 5 × 10^− 8^)

**Fig. 2:**
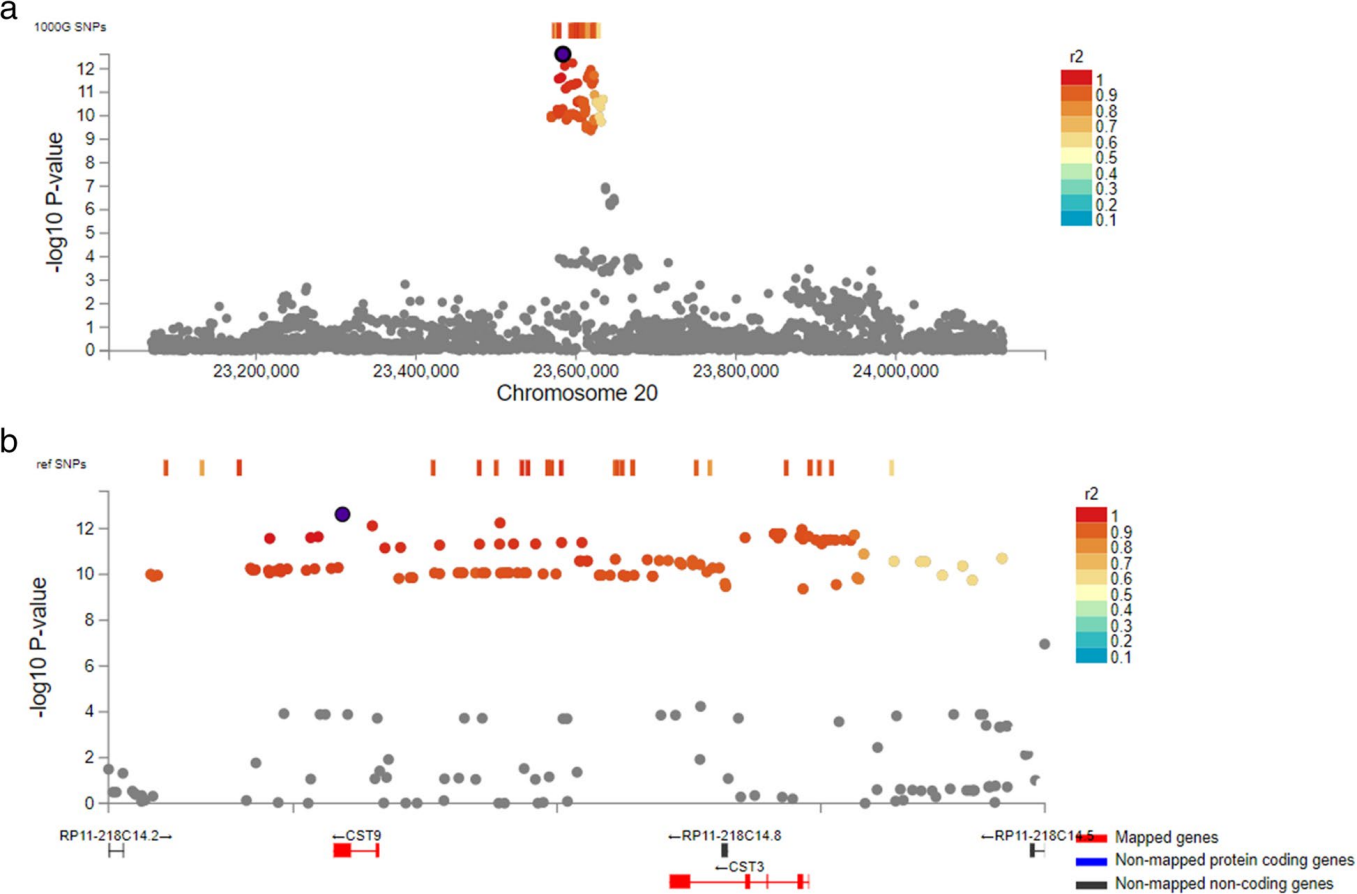
Regional plot for rs13037490 on chromosome 20 associated with eGFR. The –log10-transformed P values are plotted against the genomic position. Dark purple encircled with black represents rs13037490; gray represents SNPs below the significance level; red circles represent significant SNPs; and circles with color ranging from different shades of red to blue indicates the range of pairwise *r* ^2^ value with the top lead SNP (rs13037490). **A** Regional plot, **B** zoomed in on **A**. Mapped genes, non-mapped protein coding genes, and non-mapped non-coding genes are represented by red, blue and dark gray respectively

After performing the GWAS analyses, 19 loci were significantly (*P* value < 5 × 10^− 8^) associated with fasting glucose, HbA1c, QUICKI, and eGFR (see Fig. 1 and Additional file 1: Fig S15-S32). The list of lead SNP is given in Table 1. eGFR had the most genome-wide significant SNP associations, with 107 significant SNPs within chromosome 20 (see Fig. 2), all showing an increasing effect of the minor allele on eGFR. The strongest association among these variants was seen for rs13037490 (*P* value = 2.4 × 10^−13^, see Fig. 2), a 3′ UTR variant localizing to *CST9* in a previously reported locus [21]. For the same trait, we found an additional independent genome-wide significant variant (*P* value = 4.6 × 10^−08^), an intronic variant in *ERBB4* (see Additional file 1: Fig. S26). Nominal (1 × 10^−7^ > *P* < 0.05) signals for four traits (fasting glucose, ISI, CIR, and HOMA-B) in MEDIM were listed as “highly ranked” variants in GWAS Catalog, suggesting that these signals are likely to be false negative in MEDIM owing to insufficient statistical power (see Additional file 2: Table S2). Quantile–quantile (Q-Q) plots illustrating significantly associated traits are shown in Additional file 1: Fig S33 for eGFR, HbA1c, fasting glucose, and QUICKI.

**Table 1:** Lead SNPs (with genome-wide significant signals) associated with FG, HbA1C, QUICKI, and eGFR

Trait	Lead SNPs	Gene/nearest gene	Chr	Position^a^	Effect allele	Beta	SE	*P* value
eGFR	rs73985808	*ERBB4*	2	212565932	A	− 0.08	0.01	4.6 × 10^−08^
eGFR	rs13037490	*CST9*	20	23583725	C	0.08	0.01	2.4 × 10^−13^
FG	rs11120828	*CAMTA1*	1	7122846	A	0.14	0.02	1.6 × 10^−10^
FG	rs76150693		2	228526473	G	0.33	0.042	4.4 × 10^−08^
FG	rs79451541	*NDUFA10*	2	240929777	A	1.03	0.18	1.2 × 10^−08^
FG	rs74591871		3	20241645	A	0.196	0.036	4.9 × 10^−08^
FG	rs78223279	*TRIO*	5	14397518	A	0.68	0.19	8.3 × 10^−09^
FG	rs115873798	*WWC1*	5	167759448	G	0.25	0.04	3.4 × 10^−08^
FG	rs73231408		8	24930722	T	0.75	0.12	2.2 × 10^−08^
FG	rs147360587	*TRAPPC9*	8	141360595	C	0.23	0.04	5.6 × 10^−09^
FG	rs143653828	*SH3GL2*	9	17580151	T	0.31	0.05	5.4 × 10^−11^
FG	rs77023105		12	30658931	G	0.33	0.06	3.2 × 10^−08^
FG	rs72802149	*ABCC11*	16	48197315	T	0.6	0.1	2.7 × 10^−09^
HbA1C	rs11120828	*CAMTA1*	1	7122846	A	0.16	0.02	4.4 × 10^−11^
HbA1C	rs77145902		2	121862595	T	0.28	0.05	1.9 × 10^−08^
HbA1C	rs117580692	*ME1*	6	83955807	A	1.45	0.25	2.4 × 10^−08^
HbA1C	rs72941612	PAK1	11	77059870	T	0.32	0.05	9.1 × 10^−09^
HbA1C	rs146006303	*RORA*	15	61137932	C	1.13	0.16	9.7 × 10^−12^
QUICKI	rs184544915	*METTL16*	17	2329349	C	0.49	0.08	6.3 × 10^−09^

^a^Position is according to Build 37 (GRCh37/hg19)

Several steps were undertaken to characterize the functional basis of rs13037490 (*CST9*) and rs73985808 (*ERBB4*). We began by examining variants within the LD block (*r*^2^ > 0.8, determined from the 1000 Genomes Project - internationalgenome.org) within which rs13037490 and rs73985808 reside, using HaploReg V4.1 software (HaploReg v4.1 (broadinstitute.org). Here, the functional basis of linked SNPs and small indels is ascertained using (i) annotations from Roadmap Epigenomics and ENCODE projects, (ii) sequence conservation across mammals, (iii) effect of SNPs on regulatory motifs, and (iv) the effect of index SNPs on gene transcription. Variants in high LD with rs13037490 are characterized by a variety of functional properties, with no clearly discernible pattern of causality. Thus, to narrow the search space further, we used CAUSALdb Index (http://www.mulinlab.org) to identify a 95% credible causal set, which revealed five likely causal variants, all in high LD with our index SNP rs13037490 (Additional file 2, Table S3). Because rs13037490 was in LD with this credible set but did not rank above these five variants (13th rank), it is likely that rs13037490 is not causal in and of itself but is a strong tag for the causal locus. However, the functional annotation suggests that the causal effects are likely to be primarily in testis and blood, with no clear indication of kidney-specific effects. For rs73985808, there was little evidence of function, other than that rs73985808 disrupts the binding motifs for *FOXL1*, *FOXP1*, and *PAX-4.* We also explored the possibility of using the MEDIM data for direct annotations using GARFIELD (https://www.ebi.ac.uk/birney-srv/GARFIELD/), but determined from the low estimates of certainty that the MEDIM dataset is likely to be underpowered for this purpose (data not shown), and elected not to proceed with further analyses of this nature.

## Discussion

This is one of the first analyses investigating genetic variants associated with kidney function and T2D in a Middle Eastern ancestry cohort and the first GWAS to be reported for any trait in a cohort of Iraqi ancestry. We identified a genome-wide significant signal at *CST9* for eGFR, which has also been detected in other ethnicities [21, 22]. *CST9* encodes a secreted protein believed to play a role in hematopoietic differentiation and inflammation. Variation at *CST9* has also previously been linked with cystatin C [23]. In European-ancestry populations, *CST9* variants rs1158167 (*P* value = 8.5 × 10^−09^) and rs214523146 (*P* value = 1.1 × 10^−05^) [24] have also previously been associated with serum cystatin C levels, as has rs1303830545 (*P* value = 2.2 × 10^–88^) [21] with eGFRcys.

A second key finding from this study concerns variants at *ERBB4* and kidney function. *ERBB4* is a member of the EGF receptor (EGFR) subfamily of receptor tyrosine kinases and plays a critical role developing epithelial ducts in the kidney [25]. A variant in *ERBB4* has been previously associated with diabetic nephropathy [26], and ERBB4/Erbb4 has been shown to be differently expressed in human in vitro and in murine models of renal disease [25]. The negative beta for *ERBB4* and eGFR shows that the minor allele is associated with lower eGFR (i.e., worse kidney function). Thus, the major allele (i.e., the majority of the MEDIM population) carries the protective allele. This contrasts observations in other ethnic groups, where the risk allele is the most prevalent (major) allele. It is for this reason that we conclude the Iraqi population may be genetically protected from chronic kidney disease. A previous study focusing on renal function and its association with blood pressure in MEDIM participants reported that this Iraqi cohort had better overall kidney function than native Swedes [11]. Elsewhere, a Swedish nationwide study of people with new onset T2D reported that first-generation non-Western immigrants, and Middle Eastern immigrants in particular, have lower rates of all-cause and cause-specific mortality than ancestral Swedes with new onset diabetes [27]. The current analysis indicates that the better kidney function in Iraqis compared with native Swedes may have a genetic basis, with *ERBB4* being a key locus.

The most obvious explanation for the signals observed here and not in large European ancestry GWAS cohorts is that the genetic architecture of these cohorts differs, at least at the index loci. A second explanation is that the genetic signals may be a consequence of gene-environment interactions, early life environments (intrauterine and years 1–5 postpartum) being possible examples [28].

A limitation of this analysis is the lack of an Iraqi (or ethnically-proximal) replication cohort, which is not currently available. The importance of using a Middle Eastern cohort for replication studies can be observed when trying to replicate the strongest common variant signals for T2D. For example, rs7903146 and rs12255372, localizing to *TCF7L2*, have been widely replicated across many ethnic groups including American Indians [29], Japanese [30], South Asians [31, 32], Pakistanis [33], Afro-Caribbeans [32], and Europeans, but not in Middle Eastern cohorts [34]. Indeed, barely a handful of loci identified in European ancestry populations for metabolic traits have been replicated in Middle Eastern cohorts [35–37]. The genetic discordance between Middle Eastern and non-Middle Eastern cohorts, observed here for the *ERBB4* signals, further underscores the importance of replication studies being performed in cohorts from the Middle East.

Nevertheless, in the absence of an ethnically proximal replication cohort, we looked up the strongest signals from the current analysis in GWAS Catalog (European ancestry cohorts) to determine if any published findings exist that support those reported here. Although this process has its limitations, most notably that GWAS Catalog only includes genome-wide significant results and does not include data from Middle Eastern cohorts, some of our strongest findings correspond well with those reported there. We also used the Type 2 Diabetes Knowledge Portal [38] to determine if any of our strongest hits are associated with any sub-genome-wide significant signals in European-ancestry cohorts, which we also found some evidence of, suggesting that the Iraqi population, and the features of its genome, may be more powerful for genetic discovery than European ancestry cohorts, at least for kidney function traits.

There are currently no publicly available imputation panels for people of Iraqi ancestry. When comparing patterns of genetic variation in this Iraqi cohort with other ethnicities for whom reference panels are available, it was determined that the most proximal of these was the European ancestry panel. While this is unlikely to be adequate for imputation of rare variants, for common variant imputation, which is the focus of this paper, it yields acceptable predictions of missing variants. Regardless, the region where the strongest signals were detected in this study includes > 100 variants, the majority of which were directly genotyped. In this case, imputation has no bearing on the reliability of the signal, as the signal is primarily driven by non-imputed SNPs.

Even though the signals detected for the glycemic traits are not accompanied by replication studies, most of the mapped genes had literature evidence for their involvement in the pathogenesis of T2D or glucose metabolism. For instance, *CAMTA1* was associated with T2D in a French population [39]. Several studies highlight the involvement of *CAMTA1* in the development of mature functional cells of islets as well as in regulating beta-cell insulin content and secretion. *NDUFA10* codes for the enzyme 42 kDa complex I and is involved in oxidative phosphorylation (OXPHOS) inside the mitochondria. This gene, along with other OXPHOS genes, was found to be downregulated in pancreatic islets of T2D patients and was implicated in the development of impaired glucose-stimulated insulin secretion [40, 41]. *TRIO* (Rho guanine nucleotide exchange factor), which was associated with fasting glucose, plays a role in cell migration and growth through the actin cytoskeleton’s reorganization. Dufurrena et al. recently demonstrated *TRIO*’s active role in the regulation of glucose responsiveness and proinsulin secretion [42]. *SH3GL2* (SH3 Domain Containing GRB2 Like 2, Endophilin A1) is known for its active role in lipid binding and lipid tube assembly. In 2012, *SH3GL2* was added to the list of a candidate genes for T2D, which might affect islet function [43]. *ABCC11* codes for ABC proteins, which transport molecules across extra- and intra-cellular membranes, including glucose and other sugars. A variant in *ABCC11* (ATP binding cassette subfamily C member 11) gene was associated with fasting glucose in a meta-analysis of 13 genome-wide association studies [44]. *ME1* codes for the cytosolic malic enzyme of pancreatic β cells. *ME1* enzyme links the glycolytic and citric acid cycles. This gene is known to be highly expressed when dietary carbohydrate intake is elevated and is believed to actively enhance insulin secretion [45]; although its role in insulin secretion is contested [46]. *PAK1* encodes for p21 (RAC1) activated kinase 1 proteins, which is an effector that connects the RhoGTPases to the cytoskeleton and nuclear signaling. *PAK1* is involved in the second phase of glucose-stimulated insulin secretion [47] and Islets from individuals with T2D have been found deficient in *PAK1* protein expression when compared with islets of individuals without diabetes [48, 49]. *RORA* is a member of a nuclear hormone receptor that regulates gene expression. This gene regulates the transcription of genes, which are crucial for regulation of glucose metabolism. *RORA* has been identified as a transcriptional activator of insulin [50] and may impact T2D risk through numerous pathways [51].

Although the sample size used here is small relative to many contemporary GWAS, it is evidentially adequately powered to detect signals across multiple traits and is similar in size to early GWAS cohorts. Nevertheless, the nature of the MEDIM cohort requires the use of special statistical methods to account for cryptic relatedness and family structure, which may have diminished statistical power. Accordingly, it is likely that type 2 error (false-negative rates) will be high in the current analysis, motivating future studies in larger Iraqi cohorts.

## Conclusion

This is the first GWAS to be reported within an Iraqi population. Despite its relatively small sample size, we identified novel variants associated with kidney function, glycemic control, and insulin action. The apparent genetic protection from kidney dysfunction in this cohort may help explain why people from Iraqi appear to have better kidney function than people of European ancestry.

## Supplementary Information

**Additional file 1: Fig. S1.** Flow chart for eligible study participants. **Fig S2.** Two-dimensional plots for multidimensional scaling of MEDIM participant and 1000 genomes populations. The distances between points reflect the genetic similarity. Y axis = 2nd MDS component and x axis = first MDS component. **Fig S3.** Histogram of pairwise genetic relatedness (PI-HAT) values calculated for all pairs in MEDIM participants. **Fig. S4 – Fig. S14.** Manhattan plot illustrating the −log10 (*p* values) from the MEDIM GWAS for all the 11 traits. **Fig. S15– Fig. S32**. Regional plot for all significantly associated Locus. **Fig. S33.** Quantile–quantile (Q-Q) plots of significantly association traits. Red line denotes null hypothesis (X=Y), x-axis= observed −log10 [P] & y-axis = Expected log10 [P]. (A) eGFR, (B) HbA1C, (C) Fasting glucose and (D) QUICKI.

**Additional file 2: Table S1.** Demographic and clinical characteristics of the MEDIM participants. **Table S2.** Top 20 hit SNPs in GWAS catalog found in MEDIM GWAS. **Table S3.** Characterization of the functional basis of rs13037490 (CST9) assessing CAUSALdb Index (http://www.mulinlab.org) to identify a 95% credible causal set.

## Data Availability

Access to data will be determined on a case-by-case basis, after careful evaluation of whether a given data analysis proposal is consistent with the informed consent and ethics approval for the MEDIM study and whether it overlaps with any ongoing research in the MEDIM cohort.

## Abbreviations

BMI: Body mass index
CKD: Chronic kidney disease
CIR: Corrected insulin response
DBP: Diastolic blood pressure
DIO: Oral disposition index
DNA: Deoxyribonucleic acid
eGFR: Estimated glomerulation rate
FG: Fasting glucose
GFR: Glomerulation rate
GWAS: Genome-wide association study
HbA1c: Glycated hemoglobin
HOMA-β: Homeostasis model assessment, beta
HOMA-IR: Homeostasis model assessment, insulin resistance
HRC: Haplotype Reference Consortium
IBD: Identity by descent
ISI: Insulin sensitivity index
LMM: Linear mixed model
LUDC: Lund University Diabetes Center
MAF: Minor allele frequency
MDS: Multidimensional scaling
MEDIM: The impact of Migration and Ethnicity on Diabetes In Malmö
OGTT: Oral glucose tolerance test
Q-Q plot: Quantile–quantile plot
QUICKI: Quantitative insulin sensitivity check index
SBP: Systolic blood pressure
SNP: Single nucleotide polymorphism
T2D: Type 2 diabetes
