# Supplementary material for: GWAS in people of Middle Eastern descent reveals a locus protective of kidney function—a cross-sectional study
Source: BMC Med. 2022 Mar 1;20:76. doi: 10.1186/s12916-022-02267-7 (PMC8886846; doi:10.1186/s12916-022-02267-7)

Figure Supplement to:

**GWAS in people of Middle Eastern descent reveals a locus protective of kidney function – A cross-sectional study**

Siham A Mohamed^1^, Juan Fernadez-Tajes^1^, Paul W Franks^1^*, Louise Bennet^1^*

^1^ Department of Clinical Sciences, Lund University, Malmö, Sweden

^2^ Clinical Research and Trial Center, Lund University Hospital, Sweden.

* These authors contributed equally

Correspondence: Paul W Franks (paul.franks@med.lu.se) and Louise Bennet (louise.bennet@med.lu.se)

**Figure S1**.Flow chart for eligible study participants

**Individuals excluded**


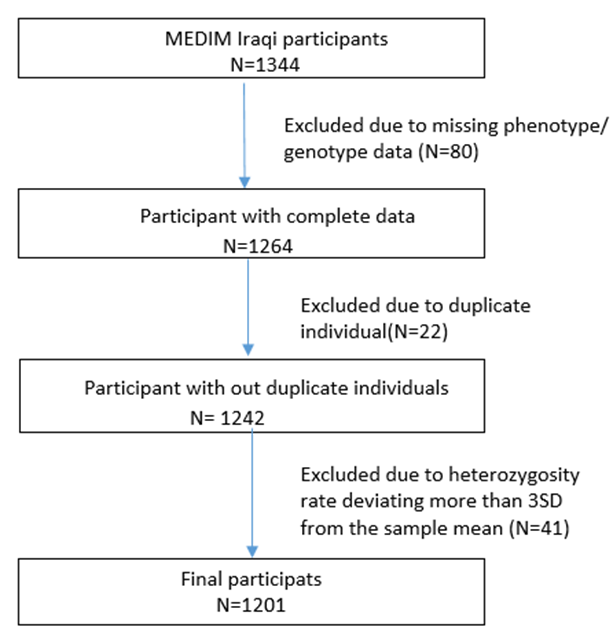


**Figure S2. Two-dimensional plots for multidimensional scaling of MEDIM participant and 1000 genomes populations. The distances between points reflect the genetic similarity. Y axis = 2nd MDS component and x axis = first MDS component.**

**Figure S2a.** There is a clear overlap between Swedish and Iraqi individuals.

+ = Native Swedes, o = Iraqi.


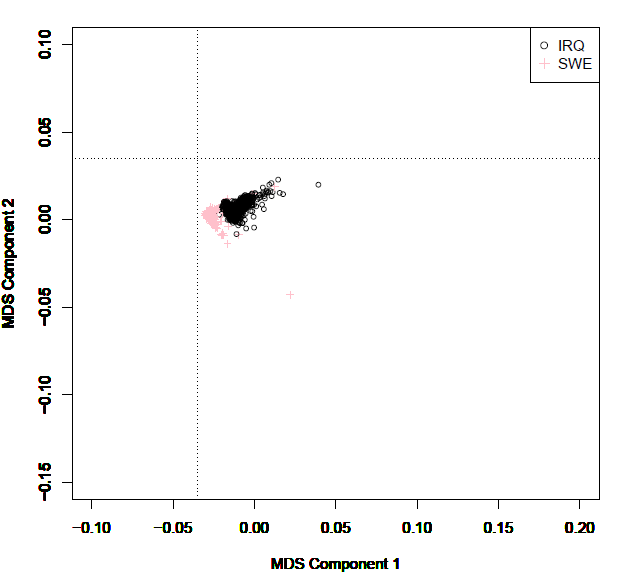


**Figure S2b** MEDIM participants clustering close to European population compared to other population, o= European, o= Asian, o= African, o= Mixed Americans and + = Iraqi and Swedish born MEDIM participants.


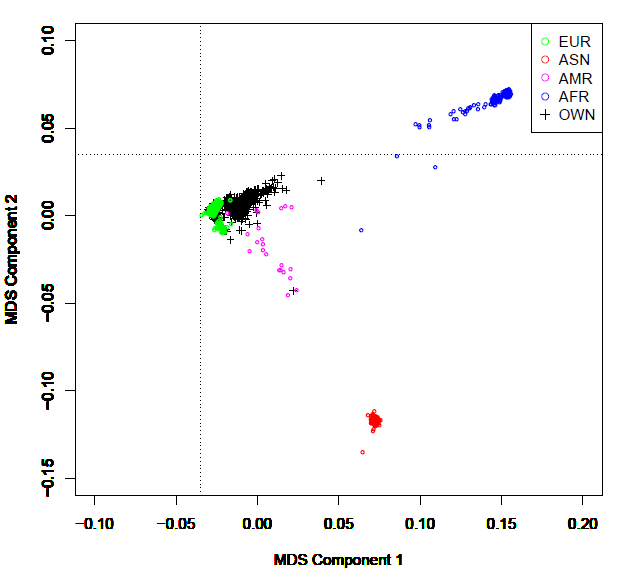


**Figure 2c.** Zoomed image of figure 2A, Two-dimensional plot for multidimensional scaling of MEDIM participant. The distances between points reflect the genetic similarity. Y axis = 2nd MDS component and x axis = first MDS component. Shows a clear overlap between Swedish and Iraqi born individuals, + = Native Swedes, o = Iraqi.


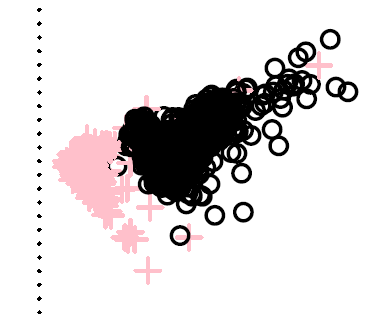


**Figure S3.** Histogram of pairwise genetic relatedness(PI-HAT) values calculated for all pairs in MEDIM participants. PI_HAT = 1 shows duplicate individuals or monozygotic twins, PI_HAT >0.20 indicates relatedness and PI_HAT value close to zero denotes not related.


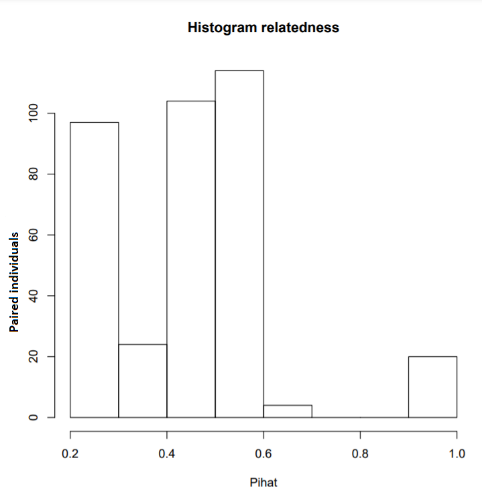


**Manhattan plot illustrating the −log10 (p-values) from the MEDIM GWAS for all the 11 traits**

**Figure S4.** Manhattan plots for the association of SNPs with BMI


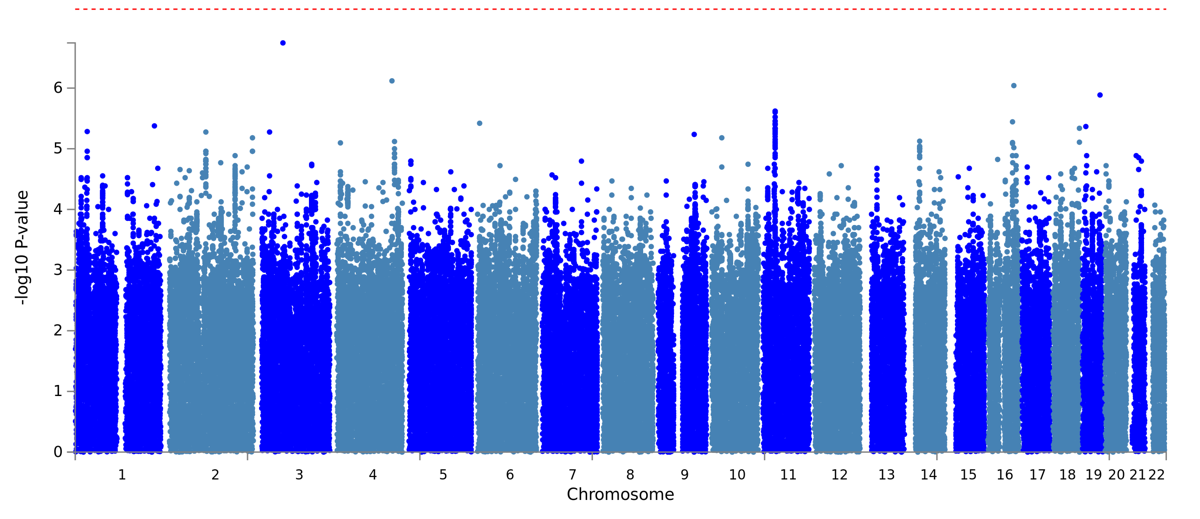


**Figure S5.** Manhattan plots for the association of SNPs with fasting glucose


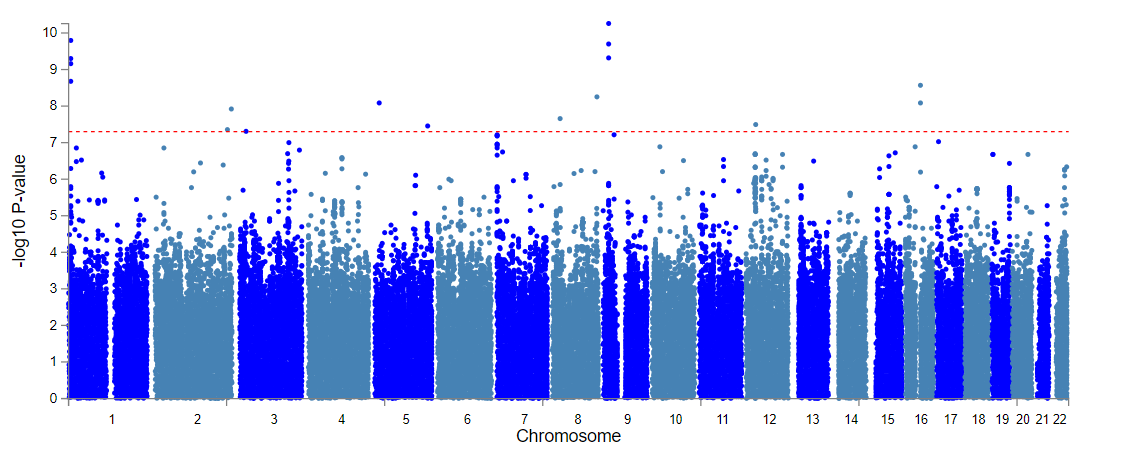


**Figure S6**. Manhattan plots for the association of SNPs with HOMA-β


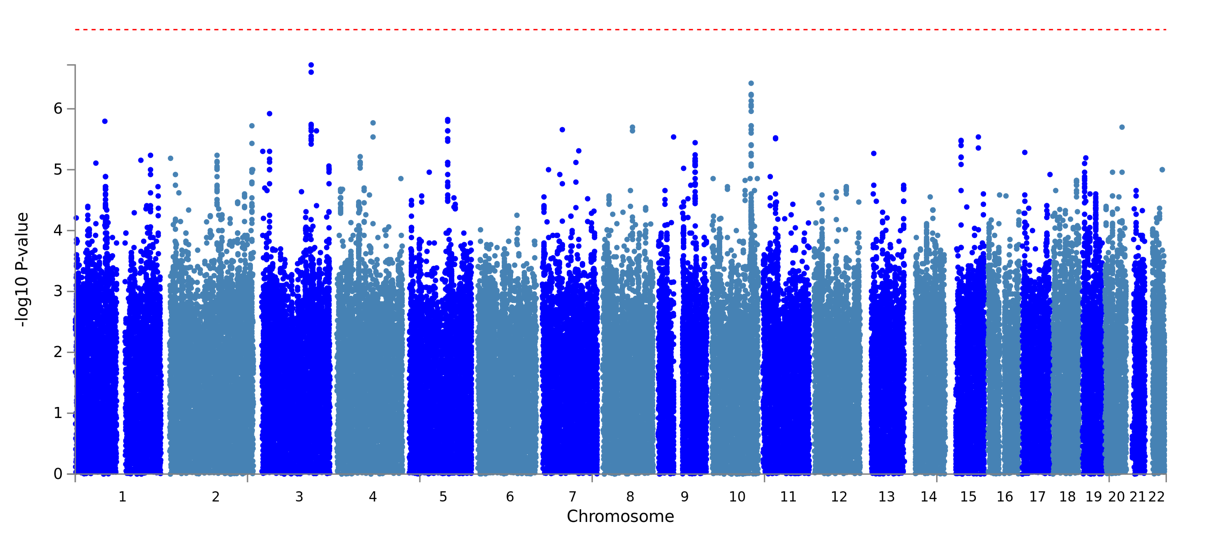


**Figure S7**. Manhattan plots for the association of SNPs with HbA1C


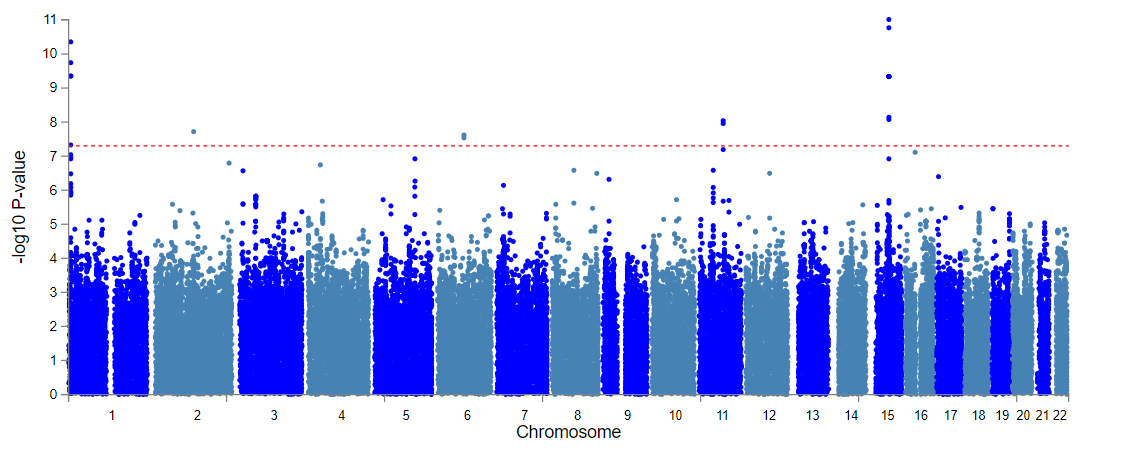


**Figure S8**. Manhattan plots for the association of SNPs with ISI


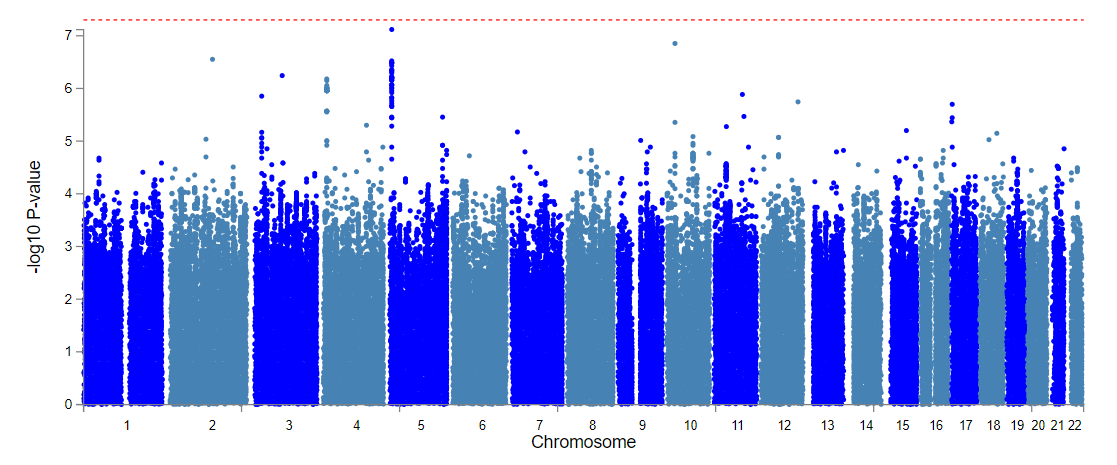


**Figure S9**. Manhattan plots for the association of SNPs with CIR


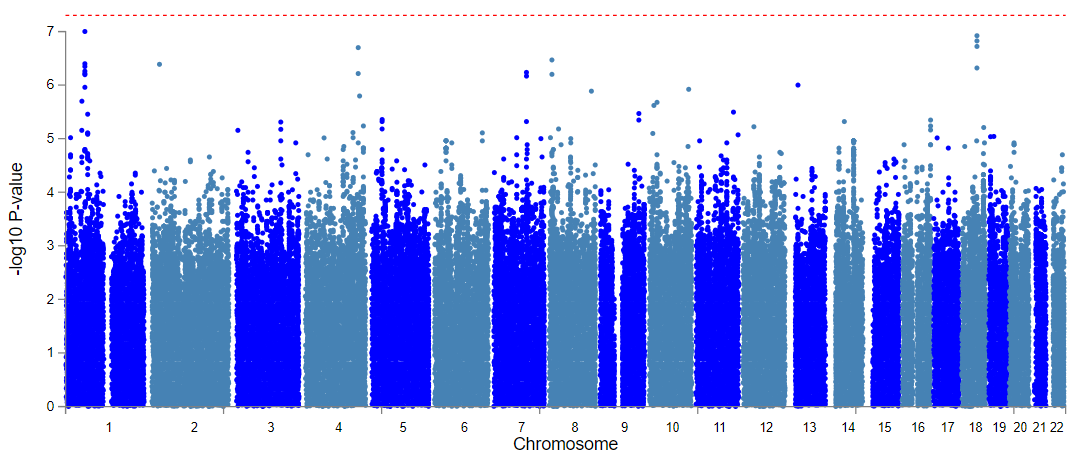


**Figure S10**. Manhattan plots for the association of SNPs with Dio


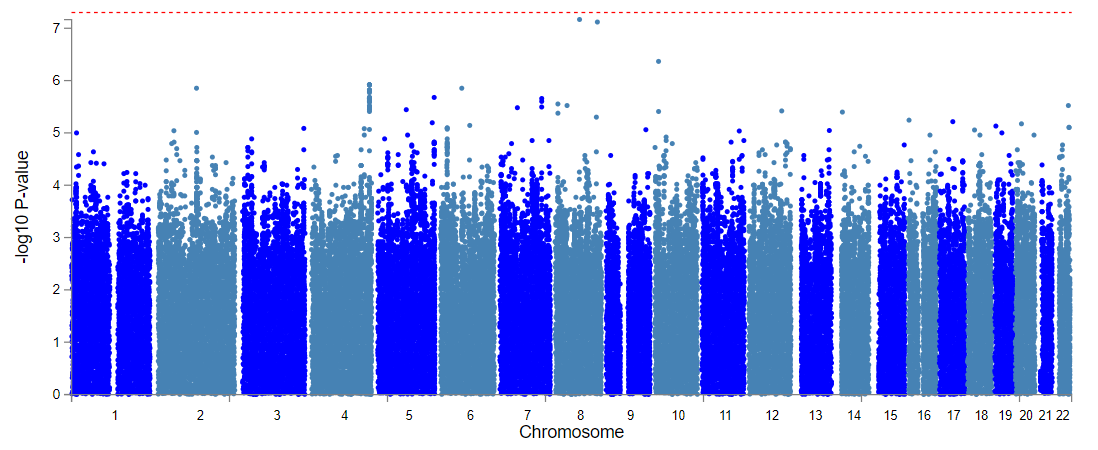


**Figure S11**. Manhattan plots for the association of SNPs with DBP


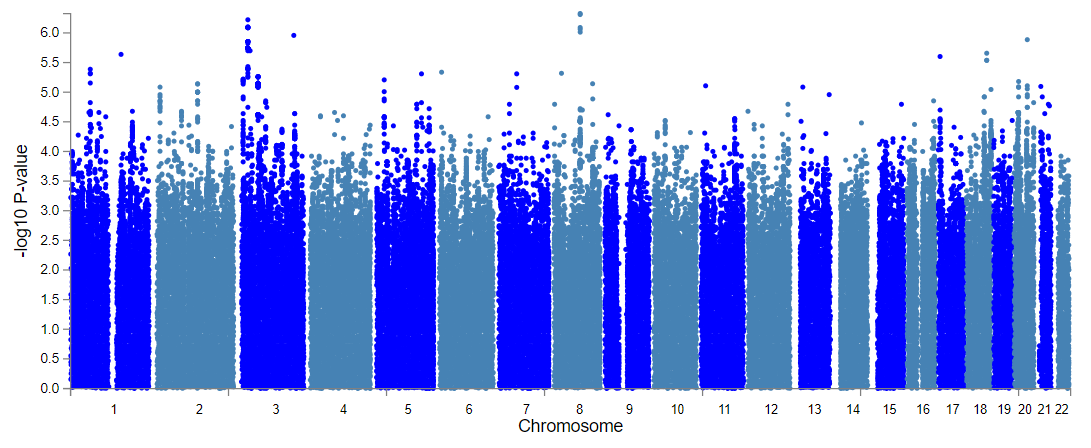


**Figure S12.** Manhattan plots for the association of SNPs with SBP


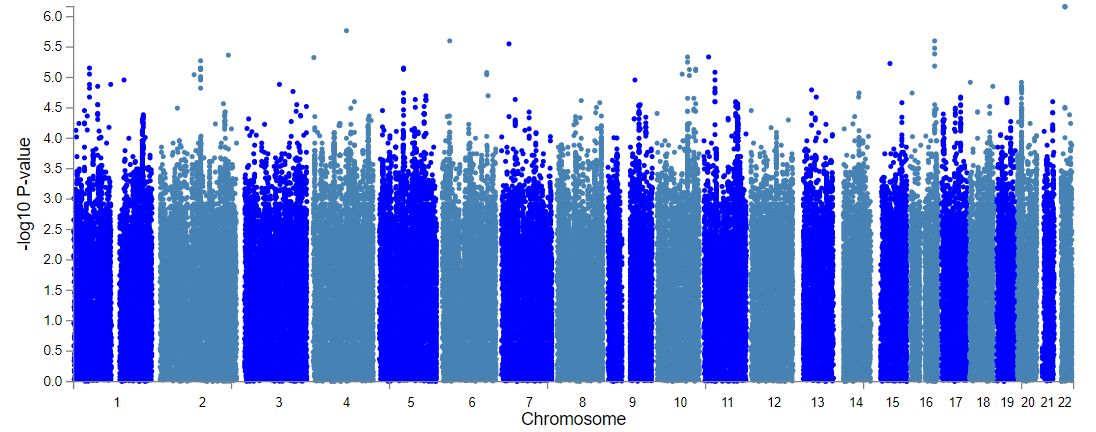


**Figure S13.** Manhattan plots for the association of SNPs with QUICKI


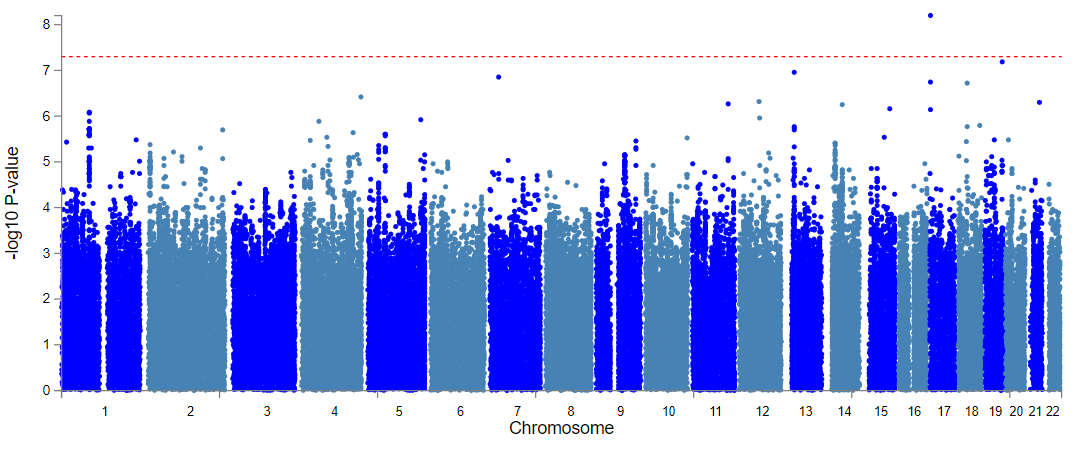


**Figure S14.** Manhattan plots for the association of SNPs with eGFR


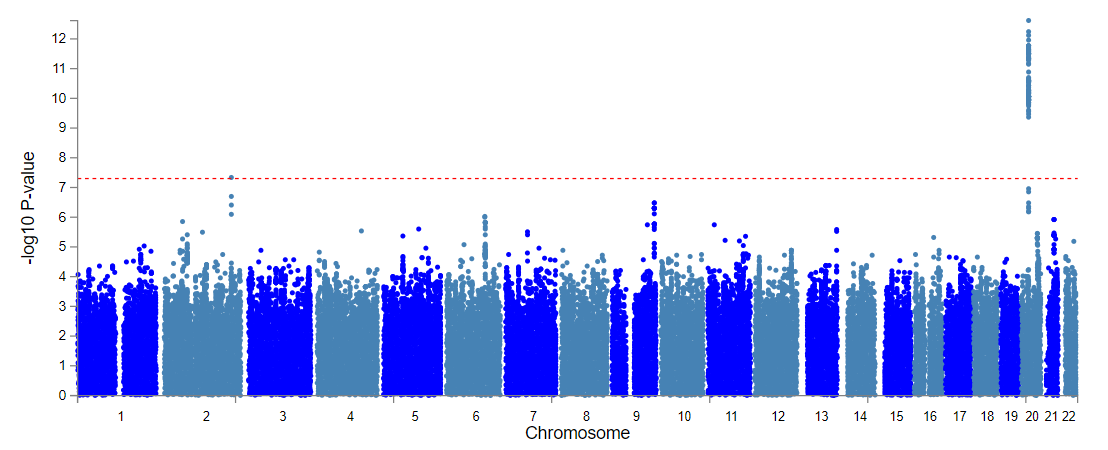


**Regional plot for all significantly associated Locus**

**Figure S15**. Regional plot for rs11120828 on chromosome 1 associated with fasting glucose. The –log10-transformed P values are plotted against the genomic position. Dark purple encircled with black represents rs11120828; grey represents SNPs below the significance level; red circles represent significant SNPs; and circles with color ranging from different shades of red to blue indicates the range of pairwise r 2 value with the lead SNP (rs11120828).


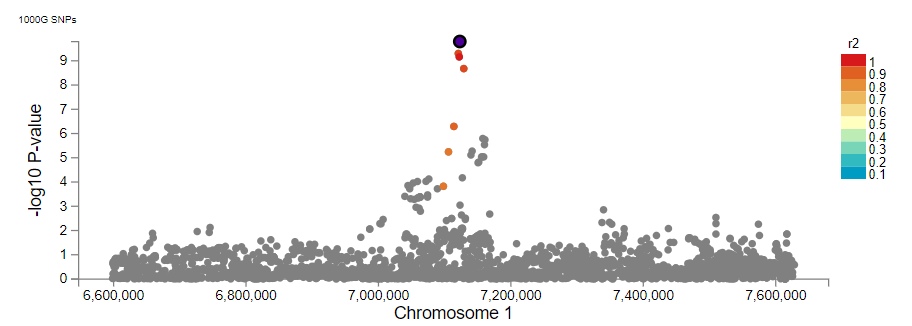


**Figure S16.** Regional plot for rs76150693 on chromosome 2 associated with fasting glucose. The –log10-transformed P values are plotted against the genomic position. Dark purple encircled with black represents rs76150693; grey represents SNPs below the significance level; red circles represent significant SNPs; and circles with color ranging from different shades of red to blue indicates the range of pairwise r 2 value with the lead SNP (rs76150693).


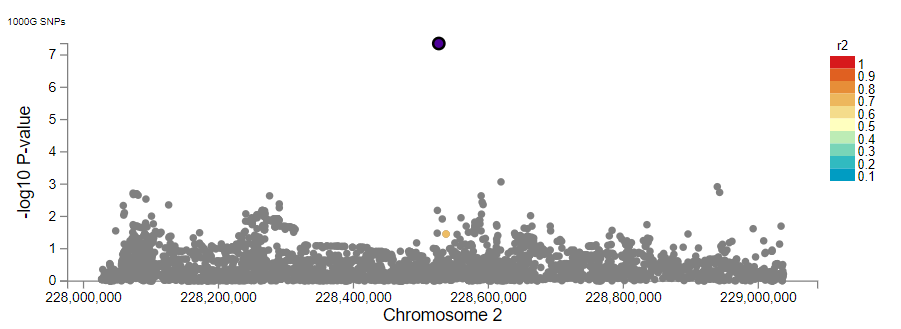


**Figure S17**. Regional plot for rs79451541 on chromosome 2 associated with fasting glucose. The –log10-transformed P values are plotted against the genomic position. Dark purple encircled with black represents rs79451541; grey represents SNPs below the significance level; and circles with color ranging from different shades of red to blue indicates the range of pairwise r 2 value with the lead SNP (rs79451541).


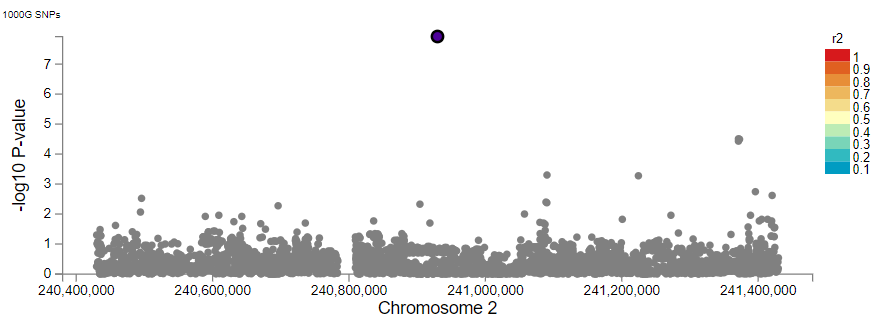


**Figure S18**. Regional plot for rs74591871 on chromosome 3 associated with fasting glucose. The –log10-transformed P values are plotted against the genomic position. Dark purple encircled with black represents rs74591871; grey represents SNPs below the significance level; and circles with color ranging from different shades of red to blue indicates the range of pairwise r 2 value with the lead SNP (rs74591871)


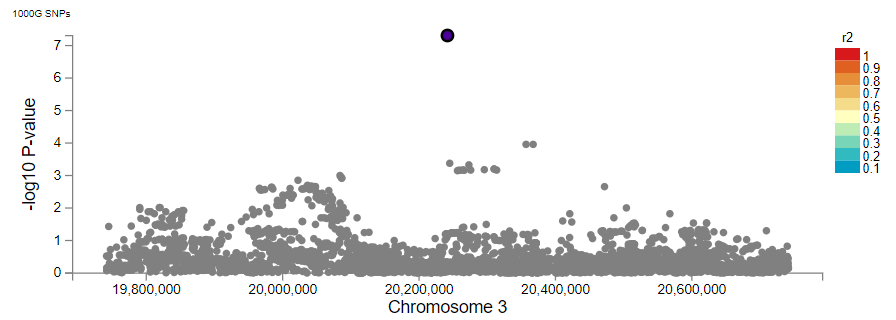


**Figure S19**. Regional plot for rs78223279 on chromosome 5 associated with fasting glucose. The –log10-transformed P values are plotted against the genomic position. Dark purple encircled with black represents rs78223279; grey represents SNPs below the significance level; and circles with color ranging from different shades of red to blue indicates the range of pairwise r 2 value with the lead SNP (rs78223279).


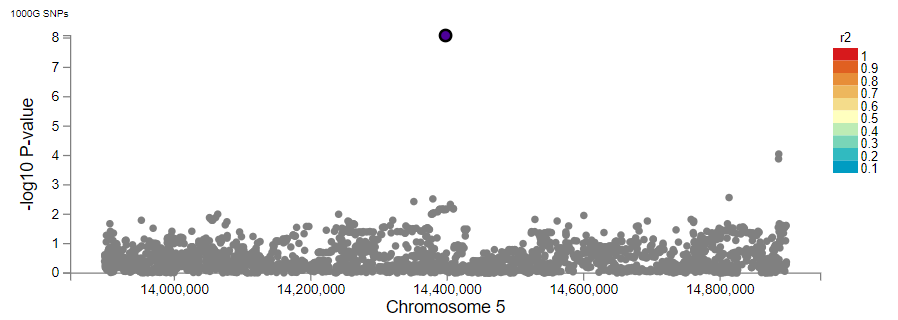


**Figure S20**. Regional plot for rs115873798 on chromosome 5 associated with fasting glucose. The –log10-transformed P values are plotted against the genomic position. Dark purple encircled with black represents rs115873798; grey represents SNPs below the significance level; and circles with color ranging from different shades of red to blue indicates the range of pairwise r 2 value with the lead SNP (rs115873798).


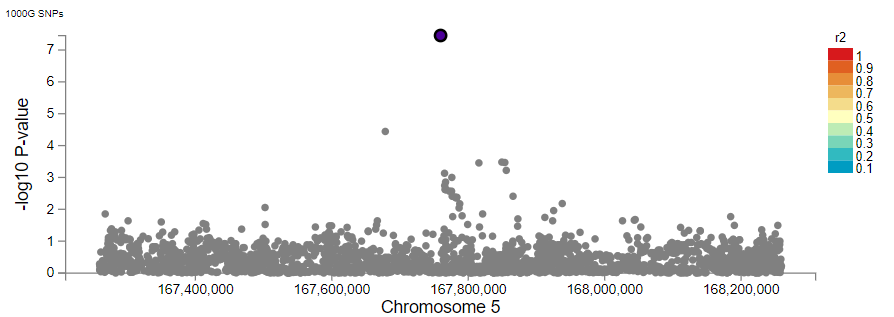


**Figure S21.** Regional plot for rs73231408 on chromosome 8 associated with fasting glucose. The –log10-transformed P values are plotted against the genomic position. Dark purple encircled with black represents rs73231408; grey represents SNPs below the significance level; red circles represent significant SNPs; and circles with color ranging from different shades of red to blue indicates the range of pairwise r 2 value with the lead SNP (rs73231408).


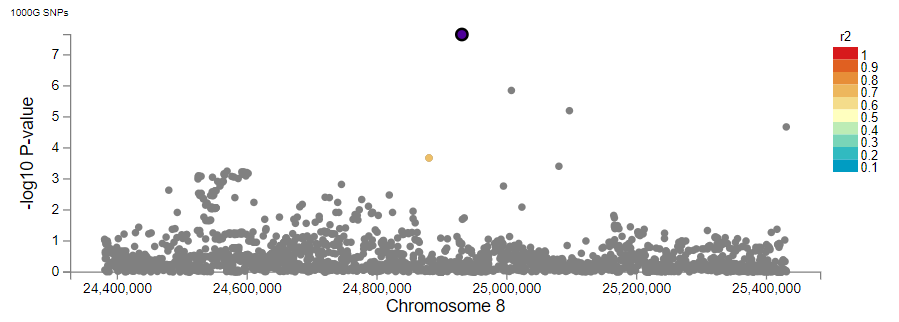


**Figure S22**. Regional plot for rs147360587 on chromosome 8 associated with fasting glucose. The –log10-transformed P values are plotted against the genomic position. Dark purple encircled with black represents rs147360587; grey represents SNPs below the significance level; and circles with color ranging from different shades of red to blue indicates the range of pairwise r 2 value with the lead SNP (rs147360587).


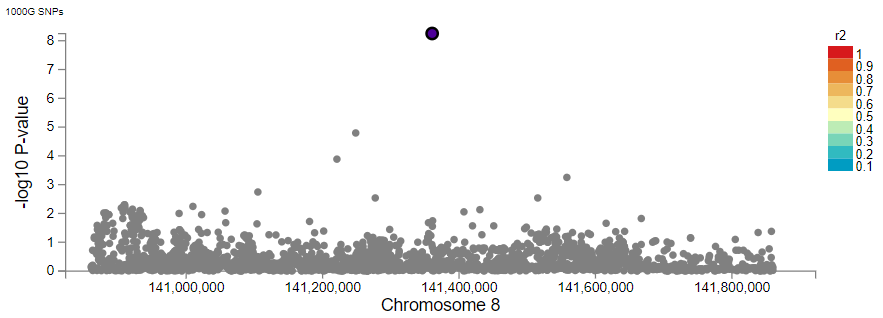


**Figure S23.** Regional plot for rs143653828 on chromosome 9 associated with fasting glucose. The –log10-transformed P values are plotted against the genomic position. Dark purple encircled with black represents rs143653828; grey represents SNPs below the significance level; red circles represent significant SNPs; and circles with color ranging from different shades of red to blue indicates the range of pairwise r 2 value with the lead SNP (rs143653828).


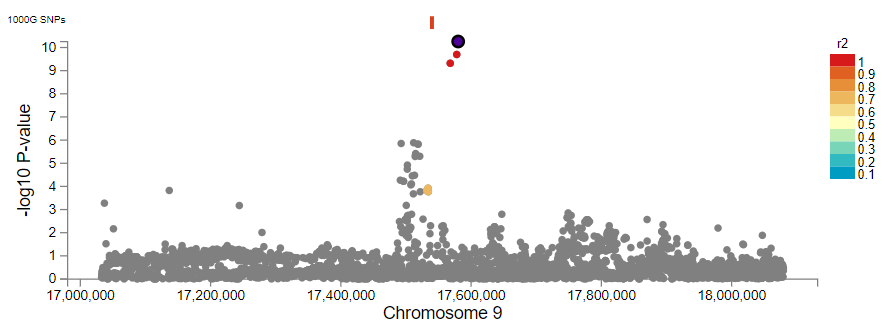


**Figure S24.** Regional plot for rs77023105 on chromosome 12 associated with fasting glucose. The –log10-transformed P values are plotted against the genomic position. Dark purple encircled with black represents rs77023105; grey represents SNPs below the significance level; red circles represent significant SNPs; and circles with color ranging from different shades of red to blue indicates the range of pairwise r 2 value with the lead SNP (rs77023105).


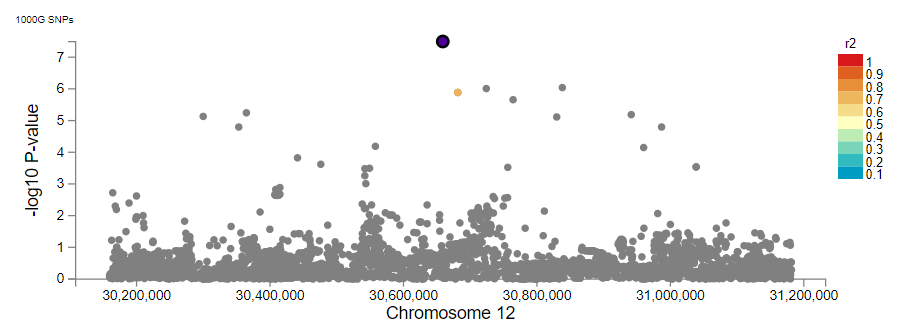


**Figure S25**. Regional plot for rs72802149 on chromosome 16 associated with fasting glucose. The –log10-transformed P values are plotted against the genomic position. Dark purple encircled with black represents rs72802149; grey represents SNPs below the significance level; red circles represent significant SNPs; and circles with color ranging from different shades of red to blue indicates the range of pairwise r 2 value with the lead SNP (rs72802149).


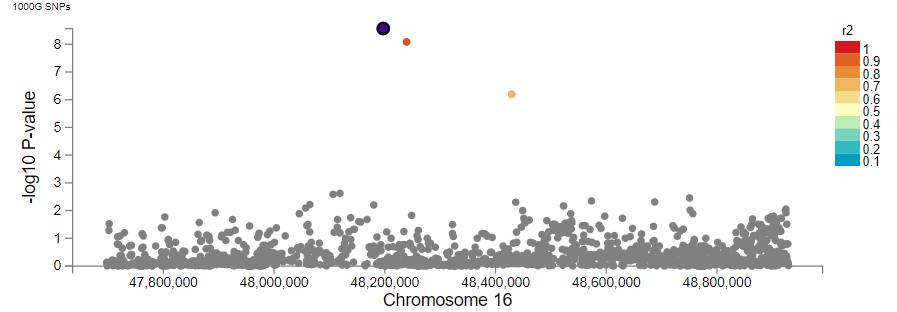


**Figure S26**. Regional plot for rs73985808 on chromosome 2 associated with eGFR. The –log10-transformed P values are plotted against the genomic position. Dark purple encircled with black represents rs73985808; grey represents SNPs below the significance level; red circles represent significant SNPs; and circles with color ranging from different shades of red to blue indicates the range of pairwise r 2 value with the lead SNP (rs73985808).


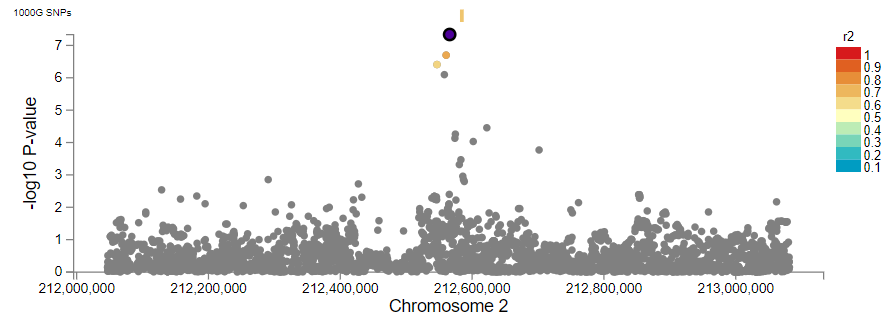


**Figure S27**. Regional plot for rs11120828 on chromosome 1 associated with HbA1C. The –log10-transformed P values are plotted against the genomic position. Dark purple encircled with black represents rs11120828; grey represents SNPs below the significance level; red circles represent significant SNPs; and circles with color ranging from different shades of red to blue indicates the range of pairwise r 2 value with the lead SNP (rs11120828).


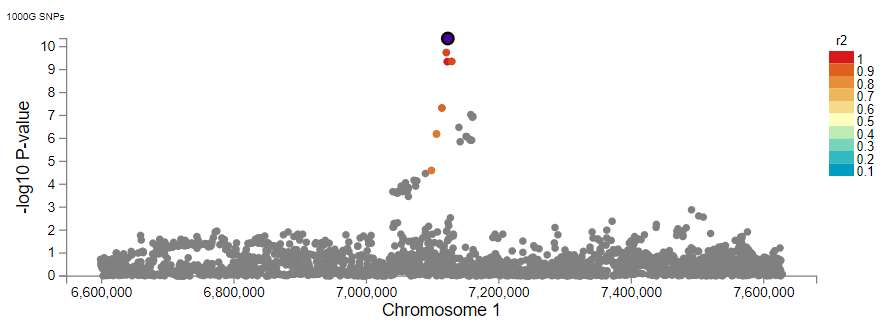


**Figure S28**. Regional plot for rs77145902 on chromosome 2 associated with HbA1C. The –log10-transformed P values are plotted against the genomic position. Dark purple encircled with black represents rs77145902; grey represents SNPs below the significance level; and circles with color ranging from different shades of red to blue indicates the range of pairwise r 2 value with the lead SNP (rs77145902).


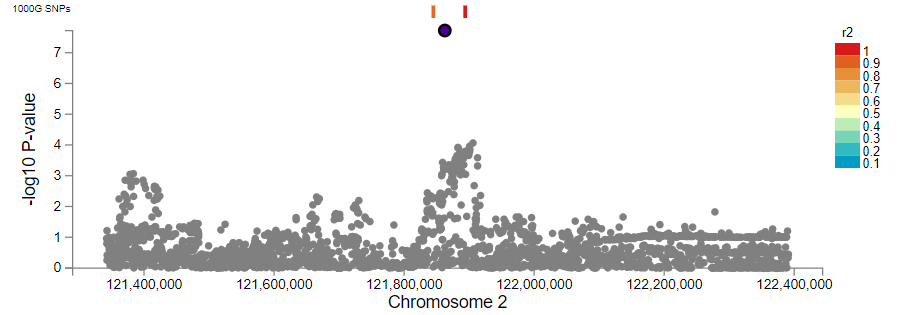


**Figure S29.** Regional plot for rs117580692 on chromosome 6 associated with HbA1C. The –log10-transformed P values are plotted against the genomic position. Dark purple encircled with black represents rs117580692; grey represents SNPs below the significance level; red circles represent significant SNPs and circles with color ranging from different shades of red to blue indicates the range of pairwise r 2 value with the lead SNP (rs117580692).


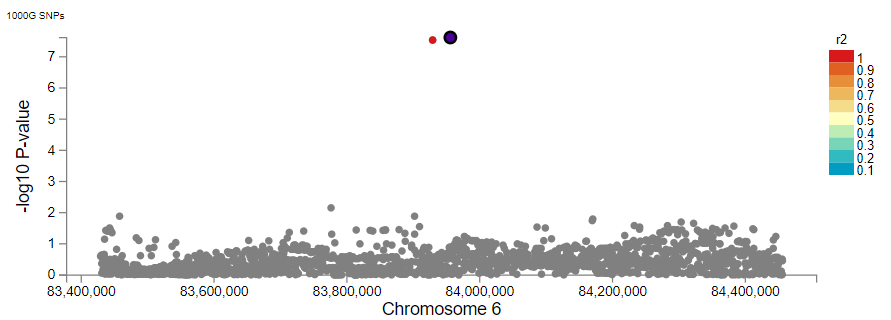


**Figure S30**. Regional plot for rs72941612 on chromosome 11 associated with HbA1C. The –log10-transformed P values are plotted against the genomic position. Dark purple encircled with black represents rs72941612; grey represents SNPs below the significance level; red circles represent significant SNPs and circles with color ranging from different shades of red to blue indicates the range of pairwise r 2 value with the lead SNP (rs72941612).


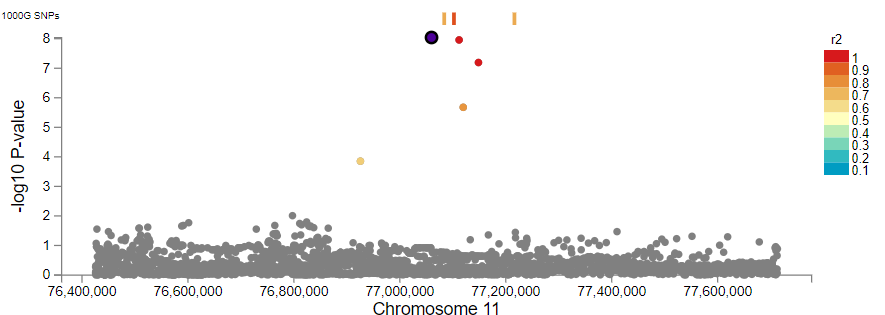


**Figure S31**. Regional plot for rs146006303 on chromosome 15 associated with HbA1C. The –log10-transformed P values are plotted against the genomic position. Dark purple encircled with black represents rs146006303; grey represents SNPs below the significance level; red circles represent significant SNPs and circles with color ranging from different shades of red to blue indicates the range of pairwise r 2 value with the lead SNP (rs146006303).


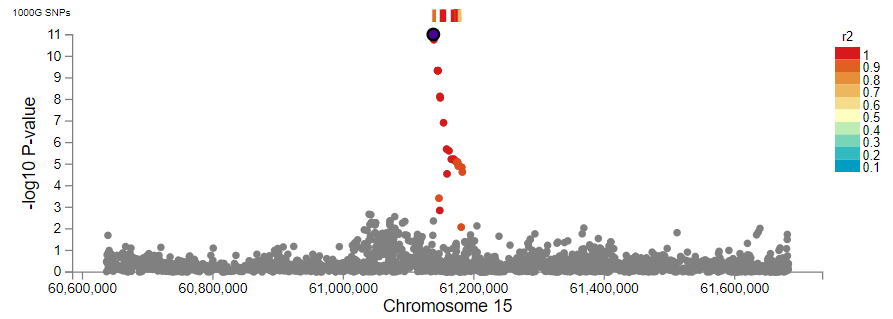


**Figure S32**. Regional plot for rs184544915 on chromosome 17 associated with QUICKI. The –log10-transformed P values are plotted against the genomic position. Dark purple encircled with black represents rs184544915; grey represents SNPs below the significance level; red circles represent significant SNPs and circles with color ranging from different shades of red to blue indicates the range of pairwise r 2 value with the lead SNP (rs184544915).


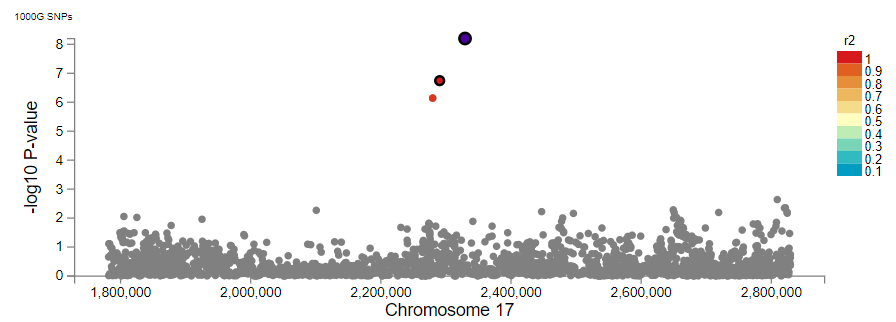


**Q-Q plot**

**Figure S33**. Quantile–quantile plots of significantly association traits. Red line denotes null hypothesis (X=Y), x-axis= observed −log10 [P] & y-axis = Expected log10 [P]. (A) eGFR, (B) HbA1C, (C) Fasting glucose and (D) QUICKI

A. B.


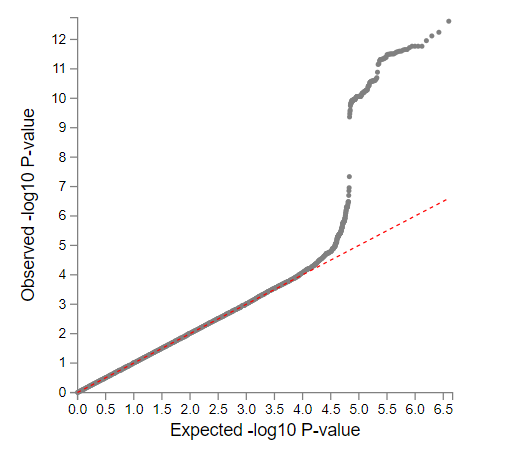

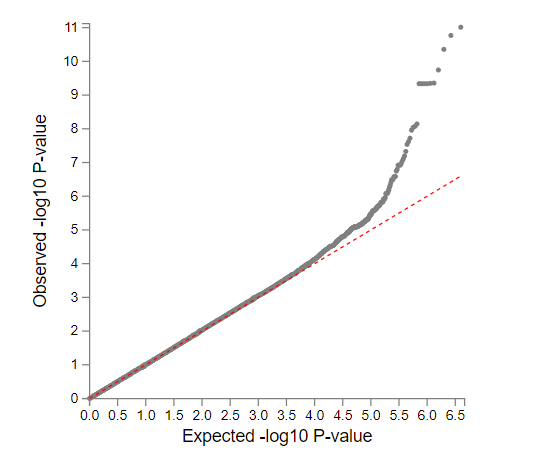


C. D.


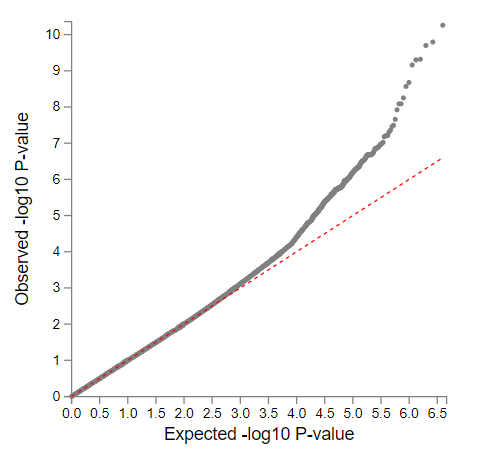

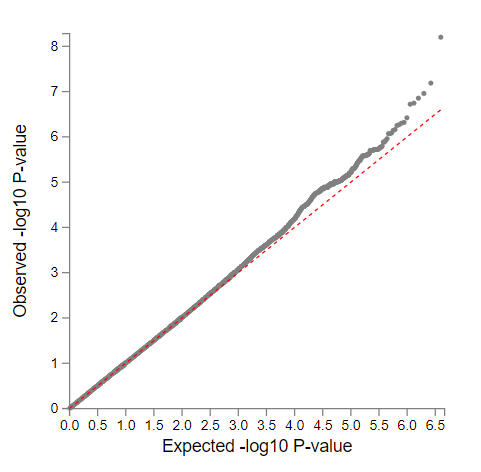

Supplement: Supplementary file 1 — Additional file 1: Fig. S1. Flow chart for eligible study participants. Fig S2. Two-dimensional plots for multidimensional scaling of MEDIM participant and 1000 genomes populations. The distances between points reflect the genetic similarity. Y axis = 2nd MDS component and x axis = first MDS component. Fig S3. Histogram of pairwise genetic relatedness (PI-HAT) values calculated for all pairs in MEDIM participants. Fig. S4 – Fig. S14. Manhattan plot illustrating the −log10 (p values) from the MEDIM GWAS for all the 11 traits. Fig. S15– Fig. S32. Regional plot for all significantly associated Locus. Fig. S33. Quantile–quantile (Q-Q) plots of significantly association traits. Red line denotes null hypothesis (X=Y), x-axis= observed −log10 [P] & y-axis = Expected log10 [P]. (A) eGFR, (B) HbA1C, (C) Fasting glucose and (D) QUICKI. [file 12916_2022_2267_MOESM1_ESM.docx]
