# Supplementary material for: GWAS in people of Middle Eastern descent reveals a locus protective of kidney function—a cross-sectional study
Source: BMC Med. 2022 Mar 1;20:76. doi: 10.1186/s12916-022-02267-7 (PMC8886846; doi:10.1186/s12916-022-02267-7)
Supplement: Supplementary file 2 — Additional file 2: Table S1. Demographic and clinical characteristics of the MEDIM participants. Table S2. Top 20 hit SNPs in GWAS catalog found in MEDIM GWAS. Table S3. Characterization of the functional basis of rs13037490 (CST9) assessing CAUSALdb Index (http://www.mulinlab.org) to identify a 95% credible causal set. [file 12916_2022_2267_MOESM2_ESM.zip › Additional File 2_Dec_20thR1.docx]

Table Supplement to:

**GWAS in people of Middle Eastern descent reveals a locus protective of kidney function – A cross-sectional study**

Siham A Mohamed^1^, Juan Fernadez-Tajes^1^, Paul W Franks^1^*, Louise Bennet^1^*

^1^ Department of Clinical Sciences, Lund University, Malmö, Sweden

^2^ Clinical Research and Trial Center, Lund University Hospital, Sweden.

* These authors contributed equally

Correspondence: Paul W Franks (paul.franks@med.lu.se) and Louise Bennet (louise.bennet@med.lu.se)

**Table S1**. Demographic and clinical characteristics of the MEDIM participants

|  | Participants born in Iraq  (N=1,201)  (mean ± SD) |
| --- | --- |
| Age^a^ | 45.0 (38.1-52.4) |
| Male | 60.49% |
| Individuals with diabetes | 8.9% |
| BMI (Kg/m^2^) | 29.17 (±4.37) |
| FG (mmol/L) | 5.89(±1.43) |
| HOMA-β | 112.83(±94.46) |
| HbA1C (mmol/L) | 37.66(±9.50) |
| ISI | 92.18(±65.38) |
| CIR | 264.49(456.96) |
| Dio | 22448.6(±65032.22) |
| Quicki | 0.59(±0.13) |
| DBP (mmHg) | 77.92(±10.12) |
| SBP (mmHg) | 128.6 (±16.44) |
| eGFR | 89.75(±18.62) |

Data presented as mean (±SD), percentile or median (interquartile range).

^a^Data presented as median (interquartile range)

**Table S2** Top 20 hit SNPs in GWAS catalog (European ancestry) found in MEDIM GWAS

| Trait | SNP | GENE | Chr | Pos | MEDIM  P-value | GWAS Cat  P-value |
| --- | --- | --- | --- | --- | --- | --- |
| F. glucose | rs560887 | *G6PC2* | 2 | 169763148 | 1.9x 10^-02^ | 9 x 10^-218^ |
| F. glucose | rs10830963 | *MTNR1B* | 11 | 92708710 | 1.1 x 10^-03^ | 6 x 10^-175^ |
| F. glucose | rs780094 | *GCKR* | 2 | 27741237 | 9.2 x 10^-06^ | 3 x 10^-24^ |
| F. glucose | rs3736594 | *MRPL33* | 2 | 27995781 | 2.8 x 10^-02^ | 2 x 10^-16^ |
| F. glucose | rs1371614 | *DPYSL5* | 2 | 27152874 | 1.7 x 10^-03^ | 3 x 10^-11^ |
| F. glucose | rs7903146 | *TCF7L2* | 10 | 114758349 | 3.0 x 10^-02^ | 1 x 10^-9^ |
| F. glucose | rs6048205 | *LINC00261* | 20 | 22559601 | 2.9 x 10^-02^ | 6 x 10^-9^ |
| F. glucose | rs12243326 | *TCF7L2* | 10 | 114788815 | 1.3 x 10^-03^ | 7 x 10^-13^ |
| ISI | rs10830963 | *MTNR1B* | 11 | 92708710 | 2.7 x 10^-02^ | 4 x 10^-30^ |
| ISI | rs6013915 | *PFDN4, AL133335.2* | 20 | 52810377 | 2.5 x 10^-02^ | 2 x 10^-9^ |
| ISI | rs11187144 | *HHEX, AL590080.1* | 10 | 94469980 | 8.1 x 10^-03^ | 3 x 10^-8^ |
|  |  |  |  |  |  |  |
| CIR | rs10830963 | *MTNR1B* | 11 | 92708710 | 9.8 x 10^-04^ | 4 x 10^-30^ |
| CIR | rs11187144 | *HHEX, AL590080.1* | 10 | 94469980 | 5.6 x 10^-04^ | 3 x 10^-8^ |
| CIR | rs174541 | *FADS2* | 11 | 61565908 | 2.4 x 10^-03^ | 5 x 10^-6^ |
| CIR | rs1260326 | *GCKR* | 2 | 27730940 | 3.4 x 10^-03^ | 6 x 10^-13^ |
| CIR | rs10483182 | *Z82196.2, AL024495.1* | 22 | 35135106 | 6.4 x 10^-03^ | 8 x 10^-12^ |
| CIR | rs13422522 | *AC068138.1, AC062015.1* | 22 | 227011320 | 5.2 x 10^-03^ | 1 x 10^-11^ |
| CIR | rs4887140 | *INSYN1-AS1* | 15 | 74046663 | 1.9 x 10^-02^ | 7 x 10^-8^ |
| HOMA-B | rs1260326 | *ADCY5* | 2 | 27730940 | 3.8 x 10^-02^ | 3 x 10^-12^ |
| HOMA-B | rs4506565 | *TCF7L2* | 10 | 114756041 | 5.4 x 10^-06^ | 7 x 10^-11^ |

**Table S3. C**haracterization of the functional basis of rs13037490 (CST9) assessing CAUSALdb Index (http://www.mulinlab.org) to identify a 95% credible causal set. This revealed five likely causal variants, all in high LD with the index SNP rs13037490. (see attached exel file)
